# Variant-Related Differences in Laboratory Biomarkers among Patients Affected with Alpha, Delta and Omicron: A Retrospective Whole Viral Genome Sequencing and Hospital-Setting Cohort Study

**DOI:** 10.3390/biomedicines11041143

**Published:** 2023-04-10

**Authors:** Georgios Meletis, Areti Tychala, Georgios Ntritsos, Eleni Verrou, Filio Savvidou, Iasonas Dermitzakis, Anastasia Chatzidimitriou, Ioanna Gkeka, Barbara Fyntanidou, Sofia Gkarmiri, Alexandros T. Tzallas, Efthymia Protonotariou, Kali Makedou, Dimitrios G. Tsalikakis, Lemonia Skoura

**Affiliations:** 1 Department of Microbiology, AHEPA University Hospital, School of Medicine, Aristotle University of Thessaloniki, 54124 Thessaloniki, Greece; 2 Department of Informatics and Telecommunications, School of Informatics and Telecommunications, University of Ioannina, 47100 Arta, Greece; 3 Department of Hygiene and Epidemiology, School of Medicine, University of Ioannina, 45110 Ioannina, Greece; 4 School of Medicine, Aristotle University of Thessaloniki, 54124 Thessaloniki, Greece; 5 Institute of Applied Bioscience, Centre for Research and Technology Hellas, 6th km Charilaou—Thermi Rd., Thermi, 57001 Thessaloniki, Greece; 6 Department of Emergency Medicine, AHEPA University Hospital, 54636 Thessaloniki, Greece; 7 Laboratory of Biochemistry, AHEPA University Hospital, School of Medicine, Aristotle University of Thessaloniki, 54124 Thessaloniki, Greece; 8 Department of Electrical and Computer Engineering, University of Western Macedonia, 50131 Kozani, Greece

**Keywords:** SARS-CoV-2, COVID-19, VOC, WBC, PLT, CRP

## Abstract

During the COVID-19 pandemic, different SARS-CoV-2 variants of concern (VOC) with specific characteristics have emerged and spread worldwide. At the same time, clinicians routinely evaluate the results of certain blood tests upon patient admission as well as during hospitalization to assess disease severity and the overall patient status. In the present study, we searched for significant cell blood count and biomarker differences among patients affected with the Alpha, Delta and Omicron VOCs at admission. Data from 330 patients were retrieved regarding age, gender, VOC, cell blood count results (WBC, Neut%, Lymph%, Ig%, PLT), common biomarkers (D-dimers, urea, creatinine, SGOT, SGPT, CRP, IL-6, suPAR), ICU admission and death. Statistical analyses were performed using ANOVA, the Kruskal–Wallis test, two-way ANOVA, Chi-square, T-test, the Mann–Whitney test and logistic regression was performed where appropriate using SPSS v.28 and STATA 14. Age and VOC were significantly associated with hospitalization, whereas significant differences among VOC groups were found for WBC, PLT, Neut%, IL-6, creatinine, CRP, D-dimers and suPAR. Our analyses showed that throughout the current pandemic, not only the SARS-CoV-2 VOCs but also the laboratory parameters that are used to evaluate the patient’s status at admission are subject to changes.

## 1. Introduction

The severe acute respiratory coronavirus 2 (SARS-CoV-2) is a single-stranded RNA virus, and as such, it is subjected to not only continuous point mutations under selective pressure but also a subsequent accumulation of mutations over time [1]. Since the beginning of the coronavirus infectious disease 2019 (COVID-19) pandemic, hundreds of variants presenting various mutation combinations and different clinical characteristics have emerged [2]. According to the world health organization (WHO), SARS-CoV-2 variants can be classified into different categories according to their actual or expected clinical and/or epidemiological impact on public health (https://www.who.int/activities/tracking-SARS-CoV-2-variants accessed on 5 March 2023). Among these categories, variants of interest (VOI) are those that present characteristics that warrant continuous monitoring and further evaluation, while variants of concern (VOC) are the variants that are proven to be more virulent, more transmissible, more pathogenic and at some extent may evade treatment options and/or the immune responses due to vaccination or previous infection [3,4,5]. In order to simplify nomenclature issues for the general public and healthcare providers, the WHO has also introduced a nomenclature system based on the Greek Alphabet that is applied only to SARS-CoV-2 lineages that have played an important role during the course of the COVID-19 pandemic (https://www.who.int/activities/tracking-SARS-CoV-2-variants accessed on 5 March 2023). According to this system, the Alpha, the Beta, the Gamma, the Delta (together with Delta subvariants), and specific Omicron subvariants have formed part of the VOC category and are responsible for millions of hospitalizations and deaths worldwide.

In everyday clinical practice, specific laboratory parameters are used to evaluate the overall clinical status of patients presenting at the emergency departments of hospitals and, later on, to monitor their ongoing condition in case of hospitalization. Variations of such parameters have been deemed to predict the severity of the infection and have been correlated with clinical outcomes [6]. However, to the best of our knowledge, there is no data illustrating how specific laboratory parameters may vary among patients affected by different SARS-CoV-2 VOCs documented by full genome sequencing.

In the present study, we assess the variations of cell blood count results and common biomarkers among patients affected with Alpha, Delta, Omicron 1 and Omicron 2 upon admission.

## 2. Materials and Methods

### 2.1. Study Design

We conducted a retrospective study of 330 COVID-19 patients admitted between April 2021 and April 2022 in AHEPA University Hospital, a 700-bed tertiary care hospital in Thessaloniki, Greece. During the pandemic, the AHEPA hospital served as one of the reference hospitals for COVID-19 patients in Northern Greece. Especially during the study period, our hospital participated in the COVID-19 national molecular epidemiology surveillance program organized by the National Public Health Organization. On a weekly basis, 10–20 positive specimens were selected for sequencing, taking care to include specimens from patients of all ages, both sexes and various districts of the city area. In order to achieve successful sequencing results, positive samples proceeded for sequencing only if deemed to have adequate viral loads interpreted as mid to low-cycle threshold values (Ct). To this end, samples presenting Ct ≤ 25 by the NeuMoDxTM SARS-CoV-2 assay and samples presenting Ct ≤ 20 by the Abbott Molecular Real-Time assay were analyzed further. In the present study, patient data were included when a complete laboratory control was available upon admission, and the patient was affected by a VOC. Datasets with incomplete laboratory profiles upon admission, non-VOC infections or unsuccessful sequencing results were excluded.

All specimens were collected as part of the standard of care protocol. Upon admission, laboratory data of 330 SARS-CoV-2 VOC-positive patients were retrieved from the lab’s database, including age, gender, VOC type, date of SARS-CoV-2 detection, hematologic parameters [white blood cell count (WBC), % of neutrophils (Neut%), % of lymphocytes (Lymph%), % of immature granulocytes (Ig%)], coagulative parameters [platelet count (PLT), delta-dimers (D-dimers)], biochemical parameters [urea, creatinine, glutamic-oxaloacetic transaminase (SGOT), glutamic pyruvic transaminase (SGPT)] and inflammatory parameters [C-reactive protein (CRP), interleukin 6 (IL-6), soluble urokinase-type plasminogen activator receptor (suPAR)]. Other parameters, such as procalcitonin, K^+^, Na^+^ and PT/PTT, were excluded from the analysis due to inadequate data for these variables. Outcome data regarding hospitalization in medical wards, hospitalization in the intensive care unit (ICU) and death during hospitalization were also recovered. The clinical information and SARS-CoV-2 PCR samples were collected as part of routine clinical care. Data were extracted and analyzed with permission by the AHEPA University Hospital bioethics committee (protocol code: 8234; date of approval: 7 February 2023).

According to the dynamic nomenclature for SARS-CoV-2 lineages [7] and the Greek Alphabet nomenclature proposal by the WHO, B.1.1.7 variants were categorized as Alpha; B.1.617.2 and Delta-like subvariants AY.4, AY.5, AY.7, AY.9, AY.9.2, AY.12, AY.43, AY.46, AY.84, AY.101, AY.109, AY.114, AY.120, AY.121, AY.122, AY.125 were categorized as Delta and; BA.1, BA.1.1, BA.2 and BA.2.9 were categorized as Omicron. Comparisons of the blood tests’ values were performed between the three groups of patients affected by the Alpha, Delta and Omicron SARS-CoV-2 variants. Further analyses were conducted according to sex and age groups.

### 2.2. SARS-CoV-2 Detection

The laboratory diagnosis of COVID-19 was performed on nasopharyngeal samples by real-time PCR, using either the NeuMoDxTM SARS-CoV-2 assay (QIAGEN, Germantown, MD, USA) or the Abbott Molecular Real-Time assay (Abbott, Chicago, IL, USA). The assay selection for each case depended upon the priority given during the everyday clinical practice for the respective sample. Testing of samples with higher priority was performed using the NeuMoDxTM SARS-CoV-2 assay.

### 2.3. Whole Genome Sequencing and VOC Determination

Viral RNA was extracted from nasopharyngeal samples using the MagMAX Viral/Pathogen Nucleic Acid Isolation kit in the automated KingFisher Flex platform (Thermo Fisher Scientific, Waltham, MA, USA) according to the manufacturer’s protocol. A total of 10 μL of the extracted RNA was used for the library construction with the QIAseq FX DNA Library Core Kit and the QIAseq SARS-CoV-2 primer panel (QIAGEN, Hilden, Germany). Library quantification was performed with the KAPA SYBR FAST Universal qPCR kit (KAPA Biosystems, Boston, MA, USA), and the samples were paired-end sequenced on the NextSeq500 Illumina platform with an additional step of heat denaturation of libraries before loading. The resulting sequenced reads were 150 bp—long, and they were quality-controlled using the FastQC software v.0.11.9 (Babraham Institute, Cambridge, UK).

Raw sequencing data were first processed using Trim galore for adapter and quality trimming. The cleaned reads were then mapped to the SARS-CoV-2 reference genome (Wuhan variant, NC_045512) using Minimap2 tool with a minimal chaining score equal to 40. From this process, only the paired-end sequences were retained, while any other (unmatched, multiple mappings, etc.) were removed. The produced alignment was processed (sorting, summary statistics etc.) using the samtools/bedtools software tools suite. GATK Haplotype Caller (Variant Filtration, Select Variants) was used to detect and filter nucleotide mutations present in over 90% (allele frequency equal to 0.9) of the sequences at each position of the reference genome. The detected mutations were used to create consensus sequences for each sample (bcf tools consensus), which were used as input to the Pangolin tool for phylogenetic identification and assignment of different SARS-CoV-2 strains. Ultimately, all identified mutations were annotated using the SnpEff tool and the NC_045512.2 (v.5.0) database (https://www.ncbi.nlm.nih.gov/nuccore/NC_045512.2 last accessed on 5 March 2023).

Our high-throughput data sets have been deposited in the public repository, European Nucleotide Archive (ENA) of EMBL-EBI (accession number PRJEB44141). Sample accession numbers are provided as Supplementary Material.

### 2.4. Blood Parameters Testing

Blood cell analysis was performed on XN 1000 hematology analyzer (Sysmex, Kobe, Japan), using whole blood samples in tubes with K3EDTA (Ethylene Diamine Tetra Acetic acid) as anticoagulant. Measurements of urea, creatinine, SGOT, SGPT and CRP were done in serum samples on the DxC 700 AU instrument (BECKMAN COULTER^®^, Brea, CA, USA). The suPAR was determined by the suPARnostic^®^ TurbiLatex assay (Birkerød, Denmark) on the Cobas^®^ 8000 c702 analyzer (Roche Diagnostics, Mannheim, Germany) using K3-EDTA plasma samples. The measurement of IL-6 was performed on the Cobas^®^ e 411 (Roche Diagnostics, Mannheim, Germany) in serum samples. D-dimers were measured on the ACL TOP 750 CTS (Instrumentation Laboratory, Bedford, MA, USA) coagulation analyzer in plasma samples retrieved from whole blood, drawn into buffered sodium citrate (0.109 M, 3.2%) tubes after centrifugation at 3000 rpm for 10 min.

### 2.5. Statistical Analysis

We calculated descriptive statistics for the demographic and clinical characteristics of our patients. We performed Analysis of Variance (ANOVA) and the Kruskal–Wallis test (in case of non-normally distributed data) in order to examine the association of lab indexes and COVID-19 subtypes for the entire group of patients, and moreover, we performed the same analysis separate for male and female patients. Patients were categorized based on their age into three groups (younger than 50 years of age, 50 to 70 years and older than 70 years). Two-way ANOVA was performed to explore the interaction between age groups and COVID-19 subtypes on the values of lab indexes. Two-way ANOVA was also performed to examine possible associations between the age of the patients and the interactions between clinical outcomes and COVID-19 subtypes. Chi-square tests were performed to examine the association between COVID-19 subtypes and the clinical outcomes of interest (hospitalization, ICU, and death). T-tests (or Mann–Whitney tests for non-normally distributed data) were performed to evaluate the association between the age of the patients and the clinical outcomes. Finally, logistic regression models were performed to explore the effect of age on clinical outcomes, with and without adjusting for the different COVID-19 subtypes. All analyses were performed using IBM SPSS v.28 and STATA 14 (StataCorp. 2015. Stata Statistical Software: Release 14. College Station, TX, USA: StataCorp LP).

## 3. Results

The study group included 330 patients (171 males and 159 females) with a mean age of 55.2 years (Table 1). Overall, 140 cases of Alpha, 136 cases of Delta and 54 cases of Omicron were included. Two hundred and twenty-five hospitalizations were recorded, with 46 ICU admissions and 52 fatal outcomes. The mean values of all measured parameters are shown in Table 1.

**Figure 1:**
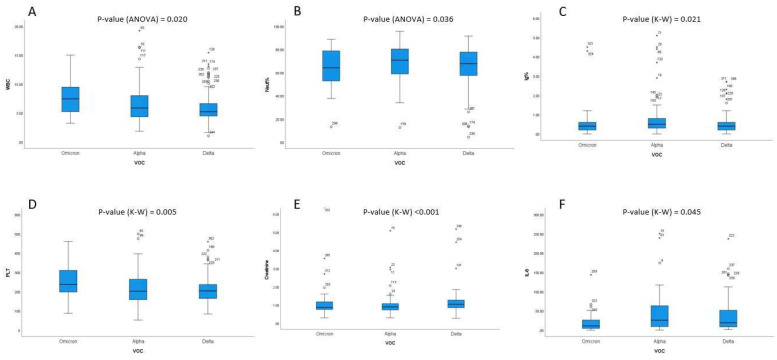
Blood count and biomarker differences among patients affected with Alpha, Delta and Omicron. (**A**) Differences in white blood cell count among the different VOC subtypes. (**B**) Differences in % of neutrophils among the different VOC subtypes. (**C**) Differences in % of immature granulocytes among the different VOC subtypes. (**D**) Differences in platelet count among the different VOC subtypes. (**E**) Differences in creatinine levels among the different VOC subtypes. (**F**) Differences in IL-6 levels among the different VOC subtypes. Stars represent extreme outliers.

Overall, WBC values were significantly differentiated among VOC subtypes (one-way ANOVA *p*-value = 0.020). More specifically, higher WBC values were found in Omicron cases (7.59) compared to Delta (6.12) (*p* = 0.017; Figure 1A). The percentage of neutrophils was significantly differentiated among VOC subtypes (one-way ANOVA *p*-value = 0.020). More specifically, Alpha showed a higher % of neutrophils (69.6) than Omicron (63.6, *p* = 0.047, Figure 1B). Moreover, % of immature granulocytes were significantly differentiated among VOC subtypes (Kruskal–Wallis *p*-value = 0.020). More specifically, statistically significant differences between Delta (0.61) and Alpha (1.07) were observed (*p* = 0.027, Figure 1C). PLT values were also significantly differentiated among VOC subtypes (Kruskal–Wallis *p*-value = 0.005). Omicron presented higher PLT counts (247) than Alpha (219, *p* = 0.018) and Delta (224, *p* = 0.005) as well (Figure 1D). Furthermore, creatinine levels were also significantly differentiated among VOC subtypes (Kruskal–Wallis *p*-value < 0.001). Participants with the Delta subtype (5.92) had higher creatinine levels compared to participants with the Alpha (1.01) and the Omicron (1.67) subtypes (Figure 1E). Finally, statistically significant differences were observed for the IL-6 levels and the different VOC subtypes (Kruskal–Wallis *p*-value = 0.045). Participants with the Alpha subtype (48.3) had higher IL-6 levels compared to participants with the Omicron (31.9) subtype (Figure 1F).

The two-way ANOVA analysis proved that the Neut% difference was mainly due to the group of participants aged >70 y. In the group of participants aged 50–70 y, the difference of Neut% between Alpha and Omicron was almost zero. Additionally, in the younger age group (<50 y), the patients with Alpha had lower Neut% values compared to patients with Delta. We also observed that the main effect of age was statistically significantly associated with the Neut% (older patients have higher Neut% values; *p*-value < 0.001). The two-way ANOVA analysis proved that this difference occurred mainly in the patients of the Alpha subgroup. In the subgroup of Omicron, the Neut% was almost the same for patients >70 and patients 50–70 y. Similarly, in the Delta subgroup, the Neut% was almost the same for patients of 50–70 y and patients <50 y. Alpha patients also had higher Ig values (1.07) compared to Delta patients (0.61, *p* = 0.027), and Delta patients presented higher creatinine values (5.92) than both Alpha (1.01, *p* < 0.001) and Omicron (1.67, *p* = 0.038) patients.

A statistically significant (*p* < 0.001) association between SARS-CoV-2 VOC and hospitalization was found. Indeed, 44.4% of the Omicron patients admitted to our hospital needed hospitalization, whereas 75% of Alpha and 70.6% of Delta patients were hospitalized. A significant association with ICU admission (*p* = 0.002) was also found. 30% of the patients with Alpha needed ICU admission, whereas only 16% of Omicron and 10% of Delta patients were admitted to the ICU. This similarity between Omicron and Delta highlights that even though Omicron cases were less likely to be hospitalized, severe Omicron cases could end up in the ICU with similar probabilities to Delta. No association was observed between VOC and death as a final outcome.

Among male patients, those infected by Omicron had higher WBC (7.5) and PLT (233) counts than those infected by Delta (6.3, *p* = 0.01 and 217, *p* = 0.007, respectively). Delta-infected male patients had higher creatinine values (1.51) than Alpha patients (1.02, *p* = 0.003). Among females, Omicron patients had higher WBC counts (7.71) than Delta (5.88, *p* = 0.034) and lower IL-6 values (median value of 4.47) than Alpha (median value of 20.46; *p* = 0.018) and Delta (median value of 21.04; *p* = 0.016). CRP values were higher for female patients with the Delta (5.10) than for those with the Omicron (2.34, *p* = 0.03) variant.

**Figure 2:**
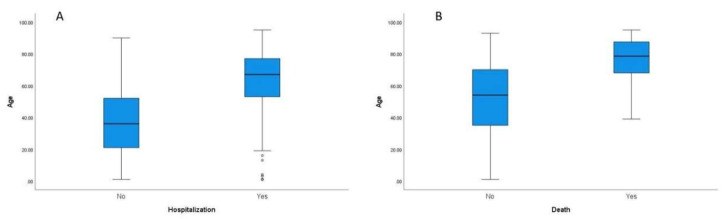
Association between age and clinical outcomes. (**A**) Association among the age of the participants and the need for hospitalization. Participants that needed hospitalization were older (mean age 63.9 vs. mean age 34.5). (**B**) Association among the age of the participants and death. Participants that died were older (mean age 76.5 vs. mean age 51.1).

A statistically significant difference between the mean age of the patients that were hospitalized compared to those that were not was found (*p* < 0.001) (Figure 2A). As expected, patients that needed hospitalization were older (mean age 63.9 vs. mean age 34.5). Logistic regression analysis showed that if age was increased by one year, the risk of hospitalization increased by 6%. Age was not statistically associated with ICU admission. There was a statistically significant difference, however, between the mean patient age and death (*p* < 0.001) (Figure 2B). Participants that died were older (mean age 76.5 vs. mean age 51.1), and according to the logistic regression results, each year of age increased the risk of death by 8.2%.

No statistically significant interactions between hospitalization and VOC on age were found. In other words, age was statistically significantly associated with hospitalization (older patients were more likely to be hospitalized), but this association did not change for different SARS-CoV-2 VOCs. That means that despite the VOC, older patients are more likely to be hospitalized. No statistically significant interactions between ICU admission and VOC on age were detected. The same was observed for death and VOC on age.

In the logistic regression analysis, however, age was statistically significantly associated with the hospitalization status of patients when adjusting for VOC. If the age was increased by one year, the risk of hospitalization increased by 6% for patients with the same VOC. Moreover, for patients of the same age, those with the Alpha VOC had a 4-fold higher risk of hospitalization compared to patients with Omicron, and those with Delta had a 3.8-fold higher risk of hospitalization compared to patients with Omicron. Age was significantly associated with death when adjusting for VOC. If the age was increased by one year, the risk of death increased by 8.3% for patients with the same VOC.

**Figure 3:**
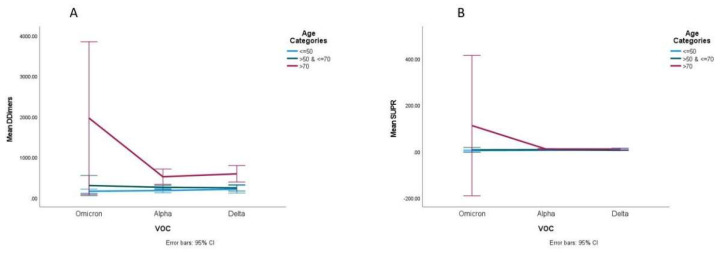
Interaction of SARS-CoV-2 variants and age on D-dimers and suPAR. (**A**) Interaction of SARS-CoV-2 variants and age on D-dimers. The D-dimers values differed significantly among the three VOC subtypes, and patients with Omicron had higher mean values of D-dimers compared to the patients with the other VOC. Two-way ANOVA analysis proved that the main reason for this difference was due to the group of participants aged >70 y, where these differences were greater compared to the other age groups. (**B**) Interaction of SARS-CoV-2 variants and age on suPAR. The suPAR values differed significantly among the three VOC subtypes, and patients with Omicron had higher mean values of suPAR compared to the patients with the other VOCs. Two-way ANOVA analysis proved that the main reason for this difference was due to the group of participants aged >70 y, where these differences were greater compared to the other age groups.

Statistically significant interactions were found among VOC and age groups on D-dimers (*p* < 0.001). We observed that D-dimers differed significantly among the three VOCs (*p*-value = 0.015). Patients with Omicron had higher mean values of D-dimers compared to patients with the other VOCs. Two-way ANOVA analysis proved that the main reason for this difference was due to the group of participants aged >70 y, where these differences were greater compared to the other age groups (Figure 3A). We also observed that the main effect of age was significantly associated with D-dimer values, meaning that older patients had higher D-dimers (*p* < 0.001). Two-way ANOVA analysis proved that the main reason for this difference was caused by patients with Omicron, where the differences between the three age groups were higher.

Statistically significant interactions among VOC and age group on the suPAR (*p* < 0.001) were observed. The suPAR values differed significantly among the three VOCs (*p* < 0.001), and patients with Omicron had higher mean values of suPAR compared to the patients with the other VOCs. Two-way ANOVA analysis proved that the main reason for this difference was (due to) the group of participants aged >70 y, where these differences were greater compared to the other age groups (Figure 3B). The main effect of age was significantly associated with the suPAR value showing that older patients had higher suPAR values (*p* < 0.001). Two-way ANOVA analysis proved that the main reason for this difference was the patients with Omicron, where the differences between the three age groups were greater, and also the Delta subgroup patients aged <50 y who had higher suPAR values compared to patients aged 50–70 y.

## 4. Discussion

Biomarkers have always been an invaluable decision-making tool in clinical practice as well as useful predictors of outcomes. Facing both the wide spectrum of clinical manifestations of COVID-19 and the unpredictability of its clinical course and complications, the research for the utility of various biomarkers at admission and during the course of the disease rendered useful results for the clinical management of the disease [8,9]. Furthermore, the variability of COVID-19 severity makes it imperative to be able to recognize those patients more at risk of deterioration or complications at admission for the benefit of both a safer triage and emergency department management of those patients and the appropriate allocation of resources [8,10]. This risk stratification should employ both clinical and laboratory criteria; thus, the need for strong evidence of their correlation emerges.

**Figure 4:**
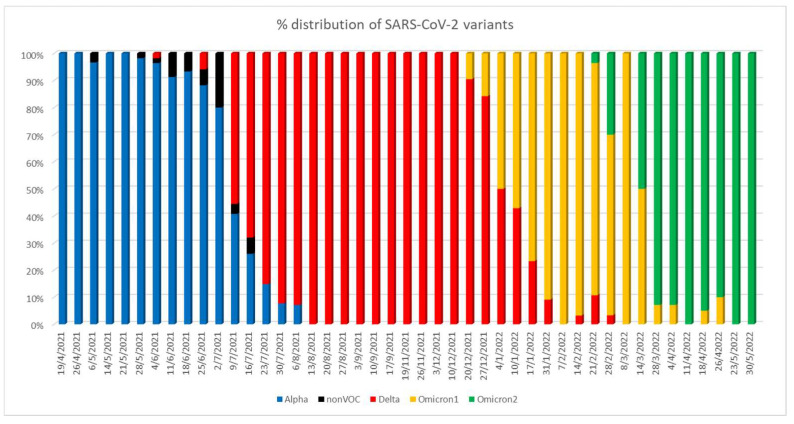
% distribution of SARS-CoV-2 variants detected in the hospital setting during the study period; nonVOC: non-variant of concern.

A novelty of our study is that we assessed various blood biomarkers among patient groups affected by different VOCs, documented by sequencing data for each patient to identify the SARS-CoV-2 variant. In fact, during the first months of the study period, Alpha was the predominant variant among positive SARS-CoV-2 samples detected in our hospital. Delta emerged on 4 July 2021 and predominated from 13 August up to mid-December. On 20 December 2021, the Omicron 1 variant (BA.1 and BA.1.1) emerged and prevailed within the range of two weeks. Delta sub-variants, however, continued to be detected at lower rates. Omicron 2 (BA.2 and BA.2.9) emerged in February 2022 and became rapidly predominant within the next months (Figure 4).

During the pandemic, it has been evident that infections with SARS-CoV-2 are associated with a wide range of symptoms. Moreover, a large study from England [11] showed changing symptom profiles associated with the emergence of different variants, similar to the changing biomarker findings in our study. The different VOCs present a number of specific mutations, some of which lead to differences in their structural proteins and, as a consequence, to their interactions with the host’s immune system. Variations in immune evasion, clinical manifestations and/or increased infectivity between VOCs are attributed to this genetic and structural variability [12,13]. Even though currently little is known in this field, the differences in clinical manifestations by the various variants could be traced to the differences in SARS-CoV-2 protein structures. In an interesting study by Goh et al. [14], it is mentioned that Omicron has a much lower N protein disorder than the other variants, which could account for its lower virulence as N is involved in rapid replication. While all VOCs have hard outer proteins (M) that could be responsible for long COVID, Omicron has among the hardest M proteins, which could be responsible for its higher D-dimer values since a harder M means that it will be more difficult for the immune system to degrade it.

At first glance, our results showed that together with the evolution of SARS-CoV-2, its interaction with the human organism is subject to changes, and this is reflected in variations regarding the values of different useful biomarkers for COVID-19. Some of our results seem to be confirmatory, like the fact that Omicron patients were less likely to be hospitalized than Alpha and Delta patients. Indeed, Alpha and Delta patients were four- and 3.8-fold more probable to be hospitalized than Omicron, respectively, and this underlines the value of molecular epidemiology surveillance to clinical practice and patient management. On the other hand, the elevated Omicron ICU admissions or the lack of significant association between VOCs and death should be considered, also taking into account that in our sample, the vaccination status and information on comorbidities were not available. The same limitations could also partially explain the fact that in our study, age was related to hospitalization and death but not to ICU admission.

Interestingly, in our study group, Omicron was related to elevated PLT counts and D-dimer values in the elderly, suggesting that thromboembolic events may be more likely to occur in such patients. According to estimates, thrombosis accounts for one in every four fatalities around the world, making it one of the main causes of mortality in the globe [15]. After 2019, the worldwide thromboembolic risk and burden have increased due to COVID-19, which has altered the entire perspective on the Venous Thromboembolic Events (VTE) rate estimation. [16]. The rate of VTEs associated with SARS-CoV-2 infection has escalated by up to 14% in the total number of COVID-19 hospitalizations [17]. Numerous studies have demonstrated that during severe COVID-19 infection, blood coagulation is greatly mobilized, which may be connected to the persistent inflammatory response brought on by the release of cytokines as a result of viral invasion [18]. The large and dynamic rise in D-dimer levels is the most important coagulation marker [18]. A rise in D-dimer levels is used to monitor the risk and progression of VTE. D-dimer levels below 0.50 ng/mL are regarded as normal, and reference values rise with pregnancy and aging [19]. D-dimer levels have been reported to increase in infection with different VOCs. Infections with both Alpha and Beta were known to considerably increase D-dimers, leading to decreased pulmonary hypoxia signaling before death as a cause of D-dimer accumulation in important organs [20,21]. Infection with the Delta variant is also considered to induce an increase in D-dimer serum levels [22]. Other studies and meta-analyses have also found that D-dimer levels were noticeably greater in COVID-19 patients than in healthy controls, and this coagulation derangement has been correlated with disease severity and higher mortality, confirming our findings [6,18,23,24,25,26,27,28]. However, contrary to our findings, a study by Shulman AH et al., 2022 carried out in Johannesburg showed that D-dimer serum levels in patients infected by the Omicron variant did not rise as much compared to those infected by the Delta variant [22]. Nonetheless, age was found to significantly contribute to the elevation of D-dimers, similar to our results [22].

SuPAR is the soluble form of uPAR, and elevated levels can be found in infections (hepatitis B, C, HIV), neoplasmatic and autoimmune diseases. It has already been shown that high levels of suPAR are observed in COVID-19 patients and especially those with severe or critical illness, who had longer hospitalization and higher demands of oxygen therapy [29]. SuPAR plays an important role in inflammation, coagulation and the interaction between endothelial cells and neutrophils. Therefore, it has been shown that hospitalized patients who had high levels >6 ng/mL of suPAR were at higher risk of developing severe respiratory failure or dying, so the suPAR upon admission was used as a prognostic biomarker [30]. We moreover found significantly elevated levels of suPAR in elderly Omicron patients compared to any other group in our study.

From the early stages of the pandemic, the need to interpret the results of fast and easy-to-perform blood tests in the context of COVID-19 severity and/or outcome was evident. For this reason, several systematic reviews aimed to collectively assess the results of various individual research works. In a systematic review by Tjendra et al. [23], several hematologic and coagulation biomarkers were found to be significantly altered, including lymphopenia (decreased CD3, CD4, CD8 T-lymphocytes), neutrophilia, increased neutrophil-to-lymphocyte ratio (NLR), thrombocytopenia, decreased eosinophil count, prolonged prothrombin time (PT), increased D-dimers, and increased fibrinogen or fibrinogen degradation products (FDP). Among these, lymphopenia, thrombocytopenia and coagulation disorders were most likely associated with severe illness. Regarding acute phase proteins and cytokines, an increase in CRP, ferritin, serum amyloid A (SAA), procalcitonin and Il-6 and a decrease in proalbumin were found to be correlated with worse progression. Additionally, the elevation of liver, muscle and renal biomarkers, including lactate dehydrogenase (LDH), creatine kinase (CK), SGOT, SGPT, blood urea nitrogen (BUN), total bilirubin and creatinine, was observed in severe cases.

In a later systematic review and meta-analysis [9], lymphopenia was associated with a threefold higher risk for severe disease and low platelet counts with adverse outcomes. The evaluation of inflammatory biomarkers showed a fourfold and sixfold higher risk for severe illness related to increased levels of CRP and procalcitonin (PCT), respectively. Higher CK, SGOT and D-dimers were associated with a threefold higher risk, whereas SGPT with 1.5-fold and LDH with a sixfold higher risk for severe disease. However, the analysis of all parameters showed significant heterogeneity among the included studies.

In a systematic review and meta-analysis regarding the role of IL-6 in COVID-19, it was shown that higher levels of IL-6 were associated with poor clinical outcomes [31]. More specifically, patients with acute respiratory distress syndrome (ARDS), ICU hospitalization, and severe symptoms had 2.9-fold higher levels of IL-6.

Another systematic review and meta-analysis investigated the role of biomarkers and attempted an estimation of threshold values that could be related to adverse clinical outcomes [28]. Hypoalbuminemia, increased CRP, D-dimers, LDH and PCT, were associated with severe disease with threshold values of 38.85 g/L, 33.55 mg/L, 0.635 μg/L, 263.5 μg/L and 0.065 ng/mL respectively.

In a systematic review and meta-analysis by Zhang et al. [18], the role of D-dimers, cytokines and lymphocyte subsets in the severity of COVID-19 was investigated. In this work, increased levels of D-dimers, cytokines, including IL-2, IL-4, IL-6, IL-10, decreased levels of B cells, NK cells and T cells, including CD3+ T cells, CD4+ T cells, CD8+ T cells and high neutrophil-to-lymphocyte ratio were associated with severe disease.

In a study by Wang et al. [32], the correlation between blood biomarkers, Omicron infection, vaccination status, and need for oxygen therapy was investigated. In the group of hospitalized patients from 2022, during the Omicron wave and with most of them being fully vaccinated, fewer patients required oxygen therapy (less than 54%) or showed poorer clinical outcomes (22.9%) or died (5.9%). Serum ferritin, LDH, CRP and plasma fibrinogen were lower during the Omicron outbreak but still played a major role in the severity of the disease and the need for oxygen therapy. In the group of fully vaccinated patients, better clinical outcomes, lower death rates and lower levels of serum ferritin, LDH, CRP and D-dimers in comparison with unvaccinated patients were observed. Based on these findings, the authors created a model for predicting the severity of the disease based on clinical and laboratory parameters.

The need for creating a risk tool based on the results of routine blood biomarkers and basic patient information that would ensure the best possible triage for COVID-19 patients led to the development of LUCAS, a COVID-19 mortality prediction calculator [33]. LUCAS uses patients’ lymphocyte count, urea and C-reactive protein, as well as their age and sex, to categorize them into different risk levels expressed as low, medium, high, and very high risk of death.

Our study presents some limitations mainly because of its retrospective nature. Obviously, the outcome and the laboratory test results of COVID-19 patients may be influenced by viral coinfections and/or superimposed bacterial and fungal infections, especially during hospitalization [34]. Even though in our study, we took into consideration only the results of tests performed at patient admission, this remains a limitation of our work. However, it is well known that the blood parameter values may vary during the course of the disease, depending on many factors, such as the severity of the infection and the patient’s response to treatment. Another limitation in our analysis is that we did not include the disease severity, comorbidities, patient ethnicity, life habits and vaccination status since these were beyond the scope of our study.

Despite these limitations, to our knowledge, this is the first study to compare blood parameters among patient groups affected by different VOCs documented by whole genome sequencing data. These findings contribute to the growing literature on COVID-19 as different variants emerge. Overall, our results showed that each VOC combines with a unique “laboratory” identity. This fact may affect the utility of laboratory markers for monitoring COVID-19 activity and progression. In the same context, the therapeutic guidelines based on laboratory values could be adjusted for distinct VOCs. Altogether, perpetual updating of knowledge about the distinctive laboratory values of each VOC is vital for clinicians setting the personalized and targeted therapy of all COVID-19 patients as an ultimate goal.

## 5. Conclusions

Our data, within the context and limitations of a real-world study, show that during the course of the pandemic, not only SARS-CoV-2 variants but also the laboratory parameters that are used to evaluate the patient’s status at admission may be subject to changes. The regular monitoring of VOIs and VOCs may provide essential information to clinicians about the circulating variants and their clinical importance. Medical doctors, however, should also be aware that the usefulness of some biomarker values may vary as variants evolve over time. Therefore, uninterrupted research and continuous medical education are needed to provide the best healthcare services to patients affected by COVID-19.

## Figures and Tables

**Table 1:** Descriptive statistics for demographic and clinical characteristics of our patients.

Characteristic	N = 330
Female, N (%)	159 (48.2)
Age in years, mean (sd)	55.2 (23.9)
VOC subtype	
Omicron, N (%)	54 (16.4)
Alpha, N (%)	140 (42.2)
Delta, N (%)	136 (41.2)
Hospitalization, N (%)	225 (68.2)
ICU, N (% of the hospitalized patients)	46 (20.4)
Death, N (%)	52 (15.8)
WBC (K/μL), mean (sd)	6.6 (3.3)
Neut%, mean (sd)	67.1 (15.7)
Lymph %, mean (sd)	22.5 (12.9)
Ig %, mean (sd)	0.8 (2.1)
PLT (K/μL), mean (sd)	226.1 (138.1)
D-dimers (ng/mL), mean (sd)	408.8 (1019.7)
Urea (mg/dL), mean (sd)	42.1 (33.7)
Creatinine (mg/dL), mean (sd)	3.1 (20.5)
SGOT (U/L), mean (sd)	43.7 (76.0)
SGPT (U/L), mean (sd)	35.5 (52.4)
CRP (mg/dL), mean (sd)	4.0 (6.0)
IL6 (pg/mL), mean (sd)	52.4 (154.1)
SUPR (ng/mL), mean (sd)	10.1 (33.0)

## Data Availability

Not applicable.

## Supplementary Materials

The following supporting information can be downloaded at: https://www.mdpi.com/article/10.3390/biomedicines11041143/s1, Sample sequence accession numbers deposited in ENA.
