# Supplementary material for: Variant-Related Differences in Laboratory Biomarkers among Patients Affected with Alpha, Delta and Omicron: A Retrospective Whole Viral Genome Sequencing and Hospital-Setting Cohort Study
Source: Biomedicines. 2023 Apr 10;11(4):1143. doi: 10.3390/biomedicines11041143 (PMC10135992; doi:10.3390/biomedicines11041143)
Supplement: Supplementary file 1 [file biomedicines-11-01143-s001.zip › biomedicines-2302383-supplementary.pdf]

Sample accession numbers deposited in the European Nucleotide Archive (ENA) (Project accession number PRJEB44141). INAB: Institute of Applied Biosciences; AHEPA: American Hellenic Educational Progressive Association; VOC: Variant of Concern; 1: Omicron 1; 2: Omicron 2; 3: Alpha; 4: Delta.

INAB sample ID	AHEPA sample ID	VOC	sampling date	sequencing date	ENA sample accession
V740	70835	3	4/15/2021	4/23/2021	SAMEA8806053
V806	78030	3	4/26/2021	4/30/2021	SAMEA8806110
V807	78260	3	4/24/2021	4/30/2021	SAMEA8806111
V808	78289	3	4/23/2021	4/30/2021	SAMEA8806112
V809	78292	3	4/23/2021	4/30/2021	SAMEA8806113
V810	78293	3	4/23/2021	4/30/2021	SAMEA8806114
V811	78296	3	4/23/2021	4/30/2021	SAMEA8806115
V812	78297	3	4/23/2021	4/30/2021	SAMEA8806116
V813	78300	3	4/23/2021	4/30/2021	SAMEA8806117
V814	78521	3	4/23/2021	4/30/2021	SAMEA14271537
V815	78523	3	4/23/2021	4/30/2021	SAMEA14271545
V816	78556	3	4/24/2021	4/30/2021	SAMEA8806120
V817	78557	3	4/24/2021	4/30/2021	SAMEA14271564
V818	78568	3	4/24/2021	4/30/2021	SAMEA14271570
V819	78569	3	4/23/2021	4/30/2021	SAMEA14271580
V820	78571	3	4/24/2021	4/30/2021	SAMEA14271590
V821	78581	3	4/24/2021	4/30/2021	SAMEA14271599
V822	78586	3	4/24/2021	4/30/2021	SAMEA14271609
V823	78611	3	4/24/2021	4/30/2021	SAMEA14271615
V824	78809	3	4/24/2021	4/30/2021	SAMEA14375467
V826	78816	3	4/24/2021	4/30/2021	SAMEA14375486
V827	78865	3	4/24/2021	4/30/2021	SAMEA14375495
V828	78878	3	4/25/2021	4/30/2021	SAMEA14375504
V902	84802	3	5/2/2021	5/10/2021	SAMEA8806200
V903	84801	3	5/2/2021	5/10/2021	SAMEA8806201
V905	84797	3	5/2/2021	5/10/2021	SAMEA8806202
V906	84440	3	5/1/2021	5/10/2021	SAMEA8806203
V907	81389	3	4/27/2021	5/10/2021	SAMEA8806204
V908	81947	3	4/28/2021	5/10/2021	SAMEA8806205
V909	81946	3	4/28/2021	5/10/2021	SAMEA8806206
V910	81636	3	4/28/2021	5/10/2021	SAMEA8806207
V912	84803	3	5/2/2021	5/10/2021	SAMEA8806209
V913	84843	3	5/2/2021	5/10/2021	SAMEA8806210
V914	84847	3	5/2/2021	5/10/2021	SAMEA8806211
V915	84849	3	5/2/2021	5/10/2021	SAMEA8806212
V916	84851	3	5/2/2021	5/10/2021	SAMEA8806213
V920	81337	3	4/28/2021	5/10/2021	SAMEA8806217
V922	81769	3	4/28/2021	5/10/2021	SAMEA8806219

V923	81773	3	4/28/2021	5/10/2021	SAMEA8806220
V924	81942	3	4/28/2021	5/10/2021	SAMEA8806221
V925	81968	3	4/28/2021	5/10/2021	SAMEA8806222
V926	81912	3	4/28/2021	5/10/2021	SAMEA8806223
V927	81466	3	4/26/2021	5/10/2021	SAMEA8806224
V928	81433	3	4/27/2021	5/10/2021	SAMEA8806225
V929	81429	3	4/27/2021	5/10/2021	SAMEA8806226
V1158	94626	3	5/13/2021	5/19/2021	SAMEA12943209
V1162	95048	3	5/13/2021	5/19/2021	SAMEA12946319
V1163	94170	3	5/13/2021	5/19/2021	SAMEA12946320
V1164	94600	3	5/13/2021	5/19/2021	SAMEA12946321
V1167	95183	3	5/13/2021	5/19/2021	SAMEA12946324
V1168	95143	3	5/13/2021	5/19/2021	SAMEA12946325
V1170	93908	3	5/13/2021	5/19/2021	SAMEA12946327
V1172	94269	3	5/13/2021	5/19/2021	SAMEA12946329
V1173	94271	3	5/13/2021	5/19/2021	SAMEA12946330
V1174	94374	3	5/13/2021	5/19/2021	SAMEA12946331
V1224	94945	3	5/13/2021	5/24/2021	SAMEA8910287
V1225	95156	3	5/14/2021	5/24/2021	SAMEA8910288
V1229	97669	3	5/17/2021	5/24/2021	SAMEA8910292
V1230	97715	3	5/17/2021	5/24/2021	SAMEA8910293
V1232	98013	3	5/18/2021	5/24/2021	SAMEA8910295
V1234	98729	3	5/18/2021	5/24/2021	SAMEA8910297
V1237	97372	3	5/17/2021	5/24/2021	SAMEA8910300
V1238	97383	3	5/17/2021	5/24/2021	SAMEA8910301
V1239	97387	3	5/17/2021	5/24/2021	SAMEA8910302
V1240	97392	3	5/17/2021	5/24/2021	SAMEA8910303
V1241	97397	3	5/17/2021	5/24/2021	SAMEA8910304
V1242	97403	3	5/17/2021	5/24/2021	SAMEA8910305
V1243	97689	3	5/17/2021	5/24/2021	SAMEA8910306
V1244	97657	3	5/17/2021	5/24/2021	SAMEA8910307
V1245	97650	3	5/17/2021	5/24/2021	SAMEA8910308
V1246	98958	3	5/18/2021	5/24/2021	SAMEA8910309
V1349	103604	3	5/25/2021	5/31/2021	SAMEA8911667
V1353	101456	3	5/22/2021	5/31/2021	SAMEA8911671
V1354	101452	3	5/22/2021	5/31/2021	SAMEA8911672
V1355	101357	3	5/21/2021	5/31/2021	SAMEA8911673
V1357	101238	3	5/21/2021	5/31/2021	SAMEA8911675
V1358	104702	3	5/9/2021	5/31/2021	SAMEA8911676
V1364	104068	3	5/25/2021	5/31/2021	SAMEA8911682
V1365	103293	3	5/25/2021	5/31/2021	SAMEA8911683
V1490	106655	3	5/29/2021	6/7/2021	SAMEA8916180
V1491	106673	3	5/29/2021	6/7/2021	SAMEA8916181
V1492	106764	3	5/29/2021	6/7/2021	SAMEA8916182
V1493	106776	3	5/29/2021	6/7/2021	SAMEA8916183
V1494	106778	3	5/29/2021	6/7/2021	SAMEA8916184

V1495	106917	3	5/29/2021	6/7/2021	SAMEA8916185
V1496	106918	3	5/29/2021	6/7/2021	SAMEA8916186
V1497	107159	3	5/29/2021	6/7/2021	SAMEA8916187
V1498	107254	3	5/29/2021	6/7/2021	SAMEA8916188
V1500	108347	3	5/31/2021	6/7/2021	SAMEA8916190
V1509	107520	4	5/30/2021	6/7/2021	SAMEA8916195
V1510	107614	3	5/31/2021	6/7/2021	SAMEA8916196
V1512	109539	3	6/2/2021	6/7/2021	SAMEA8916198
V1513	109542	3	6/2/2021	6/7/2021	SAMEA8916199
V1514	110266	3	6/3/2021	6/7/2021	SAMEA8916200
V1529	110009	3	6/2/2021	6/7/2021	SAMEA8916214
V1530	110004	3	6/2/2021	6/7/2021	SAMEA8916215
V1532	109792	3	6/2/2021	6/7/2021	SAMEA8916216
V1533	109790	3	6/2/2021	6/7/2021	SAMEA8916217
V1537	110942	3	6/3/2021	6/7/2021	SAMEA8916221
V1538	110635	3	6/3/2021	6/7/2021	SAMEA8916222
V1539	111088	3	6/3/2021	6/7/2021	SAMEA8916223
V1744	112683	3	6/7/2021	6/14/2021	SAMEA8945540
V1746	113217	3	6/7/2021	6/14/2021	SAMEA8945542
V1747	112594	3	6/7/2021	6/14/2021	SAMEA8945543
V1749	112101	3	6/6/2021	6/14/2021	SAMEA8945545
V1750	112238	3	6/6/2021	6/14/2021	SAMEA8945546
V1751	112246	3	6/6/2021	6/14/2021	SAMEA8945547
V1766	114960	3	6/9/2021	6/14/2021	SAMEA8945562
V1768	116060	3	6/11/2021	6/14/2021	SAMEA8945564
V1769	116005	3	6/11/2021	6/14/2021	SAMEA8945565
V1770	115881	3	6/10/2021	6/14/2021	SAMEA8945566
V1771	115930	3	6/10/2021	6/14/2021	SAMEA8945567
V1923	118042	3	6/14/2021	6/21/2021	SAMEA8943653
V1924	117828	3	6/14/2021	6/21/2021	SAMEA8943654
V1925	117757	3	6/14/2021	6/21/2021	SAMEA8943655
V1926	117754	3	6/14/2021	6/21/2021	SAMEA8943656
V1929	119803	3	6/16/2021	6/21/2021	SAMEA8943658
V2050	123434	3	6/23/2021	6/28/2021	SAMEA8955190
V2052	122829	3	6/22/2021	6/28/2021	SAMEA8955191
V2053	122824	3	6/22/2021	6/28/2021	SAMEA8955192
V2054	122822	3	6/22/2021	6/28/2021	SAMEA8955193
V2056	121207	3	6/18/2021	6/28/2021	SAMEA8955195
V2057	121206	3	6/18/2021	6/28/2021	SAMEA8955196
V2058	121204	3	6/18/2021	6/28/2021	SAMEA8955197
V2059	121137	3	6/18/2021	6/28/2021	SAMEA8955198
V2062	121034	4	6/18/2021	6/28/2021	SAMEA8955201
V2068	120262	3	6/17/2021	6/28/2021	SAMEA8955205
V2210	126322	3	6/27/2021	7/5/2021	SAMEA8963566
V2214	128387	3	6/30/2021	7/5/2021	SAMEA8963568
V2275	131213	3	7/4/2021	7/12/2021	SAMEA8987367

V2277	131462	4	7/4/2021	7/12/2021	SAMEA8987369
V2284	133295	3	7/6/2021	7/12/2021	SAMEA8987376
V2285	133249	3	7/6/2021	7/12/2021	SAMEA8987377
V2288	134547	3	7/8/2021	7/12/2021	SAMEA8987380
V2290	134975	4	7/8/2021	7/12/2021	SAMEA8987382
V2291	134978	4	7/8/2021	7/12/2021	SAMEA8987383
V2292	135072	3	7/8/2021	7/12/2021	SAMEA8987384
V2293	135105	3	7/8/2021	7/12/2021	SAMEA8987385
V2296	135161	4	7/8/2021	7/12/2021	SAMEA8987388
V2298	135159	4	7/8/2021	7/12/2021	SAMEA8987390
V2300	135209	4	7/8/2021	7/12/2021	SAMEA8987392
V2304	131668	4	7/4/2021	7/12/2021	SAMEA8987395
V2509	135523	3	7/9/2021	7/19/2021	SAMEA9457376
V2516	137096	4	7/12/2021	7/19/2021	SAMEA9457383
V2518	137250	3	7/12/2021	7/19/2021	SAMEA9457385
V2521	137451	4	7/12/2021	7/19/2021	SAMEA9457388
V2523	137496	4	7/12/2021	7/19/2021	SAMEA9457390
V2526	137670	4	7/13/2021	7/19/2021	SAMEA9457393
V2527	137697	4	7/13/2021	7/19/2021	SAMEA9457394
V2528	137576	4	7/13/2021	7/19/2021	SAMEA9457395
V2532	135958	4	7/9/2021	7/19/2021	SAMEA9457398
V2538	137085	4	7/12/2021	7/19/2021	SAMEA9457404
V2562	135356	3	7/9/2021	7/19/2021	SAMEA9457424
V2685	142775	3	6/28/2021	8/2/2021	SAMEA9532240
V2693	142822	4	7/20/2021	8/2/2021	SAMEA9532248
V2699	143106	4	7/21/2021	8/2/2021	SAMEA9532253
V2701	142914	4	7/20/2021	8/2/2021	SAMEA9532255
V2707	143107	4	7/21/2021	8/2/2021	SAMEA9532260
V2709	143073	3	7/21/2021	8/2/2021	SAMEA9532262
V2715	142913	4	7/21/2021	8/2/2021	SAMEA9532267
V2717	143074	4	7/21/2021	8/2/2021	SAMEA9532269
V2723	140793	4	7/17/2021	8/2/2021	SAMEA9532275
V3140	145089	4	7/24/2021	8/2/2021	SAMEA9532696
V3143	145300	4	7/24/2021	8/2/2021	SAMEA9532699
V3144	145307	4	7/24/2021	8/2/2021	SAMEA9532700
V3145	145351	4	7/24/2021	8/2/2021	SAMEA9532701
V3146	145336	4	7/24/2021	8/2/2021	SAMEA9532702
V3147	145337	3	7/24/2021	8/2/2021	SAMEA9532703
V3148	145402	4	7/24/2021	8/2/2021	SAMEA9532704
V3150	145488	4	7/24/2021	8/2/2021	SAMEA9532706
V3283	152563	4	8/4/2021	8/9/2021	SAMEA9540900
V3286	151108	4	8/2/2021	8/9/2021	SAMEA9540903
V3287	151106	3	8/2/2021	8/9/2021	SAMEA9540904
V3288	151103	4	8/2/2021	8/9/2021	SAMEA9540905
V3289	151070	4	8/2/2021	8/9/2021	SAMEA9540906
V3290	150983	4	8/1/2021	8/9/2021	SAMEA9540907

V3291	151017	4	8/2/2021	8/9/2021	SAMEA9540908
V3293	151023	4	8/2/2021	8/9/2021	SAMEA9540910
V3295	150963	4	8/1/2021	8/9/2021	SAMEA9540912
V3296	150844	4	8/2/2021	8/9/2021	SAMEA9540913
V3297	150819	4	8/2/2021	8/9/2021	SAMEA9540914
V3298	150696	4	8/1/2021	8/9/2021	SAMEA9540915
V3301	150767	4	8/1/2021	8/9/2021	SAMEA9540918
V3527	154497	4	6/8/2021	8/16/2021	SAMEA9563176
V3528	154393	4	6/8/2021	8/16/2021	SAMEA9563177
V3529	154220	4	6/8/2021	8/16/2021	SAMEA9563178
V3657	162257	4	8/17/2021	8/23/2021	SAMEA9555396
V3659	162241	4	8/17/2021	8/23/2021	SAMEA9555398
V3660	162153	4	8/17/2021	8/23/2021	SAMEA9555399
V3661	161963	4	8/17/2021	8/23/2021	SAMEA9555400
V3663	162393	4	8/17/2021	8/23/2021	SAMEA9555402
V3664	161656	4	8/17/2021	8/23/2021	SAMEA9555403
V3665	161646	4	8/17/2021	8/23/2021	SAMEA9555404
V3666	161632	4	8/17/2021	8/23/2021	SAMEA9555405
V3861	162474	4	8/18/2021	8/30/2021	SAMEA9568061
V3862	162475	4	8/18/2021	8/30/2021	SAMEA9568062
V3863	164809	4	8/21/2021	8/30/2021	SAMEA9568063
V3864	164789	4	8/21/2021	8/30/2021	SAMEA9568064
V4009	170148	4	8/29/2021	6/9/2021	SAMEA9946578
V4012	170603	4	8/30/2021	6/9/2021	SAMEA9946581
V4014	170133	4	8/29/2021	6/9/2021	SAMEA9946583
V4347	180272	4	11/9/2021	9/21/2021	SAMEA9974733
V4348	180150	4	10/9/2021	9/21/2021	SAMEA9974734
V4349	179982	4	10/9/2021	9/21/2021	SAMEA9974735
V4350	179921	4	10/9/2021	9/21/2021	SAMEA9974736
V4352	179768	4	10/9/2021	9/21/2021	SAMEA9974738
V4353	179631	4	10/9/2021	9/21/2021	SAMEA9974739
V4354	179706	4	10/9/2021	9/21/2021	SAMEA9974740
V4355	179469	4	10/9/2021	9/21/2021	SAMEA9974741
V4940	234976	4	11/13/2021	11/22/2021	SAMEA11353049
V4941	235466	4	11/14/2021	11/22/2021	SAMEA11353050
V4942	235033	4	11/13/2021	11/22/2021	SAMEA11353051
V4943	235194	4	11/13/2021	11/22/2021	SAMEA11353052
V4945	235037	4	11/13/2021	11/22/2021	SAMEA11353054
V4947	234978	4	11/13/2021	11/22/2021	SAMEA11353056
V4948	235068	4	11/13/2021	11/22/2021	SAMEA11353057
V4949	235066	4	11/13/2021	11/22/2021	SAMEA11353058
V4950	235195	4	11/13/2021	11/22/2021	SAMEA11353059
V4953	239696	4	11/18/2021	11/22/2021	SAMEA11353062
V4954	238580	4	11/17/2021	11/22/2021	SAMEA11353063
V5099	241832	4	11/21/2021	11/29/2021	SAMEA11423546
V5100	241843	4	11/21/2021	11/29/2021	SAMEA11423547

V5101	242099	4	11/21/2021	11/29/2021	SAMEA11423548
V5102	242169	4	11/21/2021	11/29/2021	SAMEA11423549
V5103	242250	4	11/21/2021	11/29/2021	SAMEA11423550
V5104	242297	4	11/21/2021	11/29/2021	SAMEA11423551
V5106	242580	4	11/22/2021	11/29/2021	SAMEA11423553
V5107	242584	4	11/22/2021	11/29/2021	SAMEA11423554
V5117	242418	4	11/22/2021	11/29/2021	SAMEA11423564
V5119	242333	4	11/13/2021	11/29/2021	SAMEA11423566
V5121	242230	4	11/9/2021	11/29/2021	SAMEA11423568
V5123	244419	4	11/2/2021	11/29/2021	SAMEA11423570
V5124	244417	4	11/23/2021	11/29/2021	SAMEA11423571
V5126	241402	4	11/19/2021	11/29/2021	SAMEA11423572
V5292	248600	4	11/29/2021	12/6/2021	SAMEA11603892
V5296	249754	4	11/29/2021	12/6/2021	SAMEA11603896
V5297	249762	4	11/29/2021	12/6/2021	SAMEA11603897
V5299	249832	4	11/29/2021	12/6/2021	SAMEA11603899
V5300	249764	4	11/29/2021	12/6/2021	SAMEA11603900
V5311	250181	4	11/30/2021	12/6/2021	SAMEA11603910
V5312	250190	4	11/30/2021	12/6/2021	SAMEA11603911
V5314	250177	4	11/30/2021	12/6/2021	SAMEA11603913
V5315	250095	4	11/29/2021	12/6/2021	SAMEA11603914
V5316	250094	4	11/29/2021	12/6/2021	SAMEA11603915
V5317	249988	4	11/29/2021	12/6/2021	SAMEA11603916
V5318	249892	4	11/29/2021	12/6/2021	SAMEA11603917
V5319	249857	4	11/29/2021	12/6/2021	SAMEA11603918
V5320	45556	4	11/29/2021	12/6/2021	SAMEA11603919
V5478	259454	4	12/8/2021	12/13/2021	SAMEA11934745
V5479	259312	4	12/8/2021	12/13/2021	SAMEA11934746
V5485	259494	4	12/8/2021	12/13/2021	SAMEA11934752
V5487	257964	4	12/7/2021	12/13/2021	SAMEA11934754
V5488	257949	4	12/7/2021	12/13/2021	SAMEA11934755
V5489	258162	4	12/7/2021	12/13/2021	SAMEA11934756
V5652	265856	4	12/15/2021	12/23/2021	SAMEA12361659
V5653	266457	4	12/16/2021	12/23/2021	SAMEA12361660
V5654	266441	4	12/16/2021	12/23/2021	SAMEA12361661
V5656	266403	4	12/16/2021	12/23/2021	SAMEA12361663
V5657	266436	4	12/16/2021	12/23/2021	SAMEA12361664
V5658	266411	4	12/16/2021	12/23/2021	SAMEA12361665
V5814	273800	4	12/24/2021	12/30/2021	SAMEA12984401
V5815	273836	4	12/23/2021	12/30/2021	SAMEA12984402
V5816	273943	4	12/24/2021	12/30/2021	SAMEA12984403
V5817	274014	4	12/24/2021	12/30/2021	SAMEA12984404
V5818	274016	4	12/24/2021	12/30/2021	SAMEA12984405
V5819	272969	4	12/23/2021	12/30/2021	SAMEA12984406
V5821	273740	4	12/23/2021	12/30/2021	SAMEA12984408
V5823	273593	4	12/23/2021	12/30/2021	SAMEA12984410

V5824	273564	4	12/23/2021	12/30/2021	SAMEA12984411
V5832	273594	4	12/23/2021	12/30/2021	SAMEA12984420
V6010	276799	4	28/12/2021	1/6/2022	SAMEA12362852
V6016	276872	4	28/12/2021	1/6/2022	SAMEA12362858
V6350	8896	1	1/12/2022	1/20/2022	SAMEA13136890
V6352	9283	4	1/12/2022	1/20/2022	SAMEA13136892
V6357	10346	4	1/13/2022	1/20/2022	SAMEA13136897
V6358	9196	1	1/4/2022	1/20/2022	SAMEA13136898
V6645	19888	1	1/24/2022	2/3/2022	SAMEA13137152
V6649	19538	1	1/24/2022	2/3/2022	SAMEA13137156
V6652	19923	1	1/24/2022	2/3/2022	SAMEA13137159
V6653	20347	1	1/25/2022	2/3/2022	SAMEA13137160
V6826	27073	1	2/1/2022	2/10/2022	SAMEA13138923
V6827	27066	1	2/1/2022	2/10/2022	SAMEA13138924
V6828	27554	1	2/1/2022	2/10/2022	SAMEA13138925
V6832	26786	1	2/1/2022	2/10/2022	SAMEA13138929
V6840	27890	1	2/2/2022	2/10/2022	SAMEA13138936
V6956	63723	1	1/9/2022	2/10/2022	SAMEA13139027
V6957	63724	1	1/24/2022	2/10/2022	SAMEA13139028
V7076	35456	1	2/9/2022	2/17/2022	SAMEA13184464
V7077	35556	1	2/9/2022	2/17/2022	SAMEA13184465
V7079	35745	2	2/10/2022	2/17/2022	SAMEA13184467
V7088	35527	1	2/9/2022	2/17/2022	SAMEA13184476
V7093	35412	1	2/9/2022	2/17/2022	SAMEA13184481
V7094	35458	1	2/9/2022	2/17/2022	SAMEA13184482
V7098	35704	1	2/9/2022	2/17/2022	SAMEA13184486
V7321	38532	1	2/14/2022	2/24/2022	SAMEA13601327
V7322	38451	1	2/13/2022	2/24/2022	SAMEA13601328
V7323	38385	1	2/14/2022	2/24/2022	SAMEA13601329
V7324	38334	1	2/13/2022	2/24/2022	SAMEA13601330
V7325	38318	1	2/13/2022	2/24/2022	SAMEA13601331
V7328	41686	1	2/1/2022	2/24/2022	SAMEA13601334
V7329	41385	1	2/1/2022	2/24/2022	SAMEA13601335
V7330	39734	4	2/14/2022	2/24/2022	SAMEA13601336
V7646	44700	2	2/21/2022	3/3/2022	SAMEA13601605
V7648	45942	1	2/22/2022	3/3/2022	SAMEA13601607
V7649	46067	1	2/22/2022	3/3/2022	SAMEA13601608
V7650	46044	1	2/22/2022	3/3/2022	SAMEA13601609
V7652	46266	2	2/22/2022	3/3/2022	SAMEA13601611
V7653	44430	1	2/21/2022	3/3/2022	SAMEA13601612
V7654	45351	1	2/21/2022	3/3/2022	SAMEA13601613
V7655	45507	1	2/21/2022	3/3/2022	SAMEA13601614
V7657	45828	4	2/21/2022	3/3/2022	SAMEA13601616
V7658	42864	2	2/17/2022	3/3/2022	SAMEA13601617
V7794	63818	1	3/2/2022	3/11/2022	SAMEA13630320
V7795	63819	1	3/1/2022	3/11/2022	SAMEA13630321

V7897	70281	2	3/21/2022	3/31/2022	SAMEA14024921
V7898	70240	2	3/21/2022	3/31/2022	SAMEA14024922
V8022	77629	2	3/30/2022	4/7/2022	SAMEA14092332
V8023	78596	2	3/30/2022	4/7/2022	SAMEA14092333
V8024	77733	2	3/30/2022	4/7/2022	SAMEA14092334
V8026	77763	2	3/30/2022	4/7/2022	SAMEA14092336
V8028	73611	2	3/25/2022	4/7/2022	SAMEA14092338
V8029	77059	2	3/29/2022	4/7/2022	SAMEA14092339
V8031	77531	2	3/29/2022	4/7/2022	SAMEA14092341
V8187	91588	2	4/14/2022	4/21/2022	SAMEA14271577
V8188	92194	2	4/14/2022	4/21/2022	SAMEA14271578
V8189	92069	2	4/14/2022	4/21/2022	SAMEA14271579
V8191	92399	2	4/15/2022	4/21/2022	SAMEA14271581
V8193	92458	2	4/15/2022	4/21/2022	SAMEA14271583
V8194	92425	2	4/15/2022	4/21/2022	SAMEA14271584
V8195	92392	2	4/15/2022	4/21/2022	SAMEA14271585
